# Development and Comparative Analysis of Hard and Soft Wheat Flour Films Enriched with Yellow and White *Chlorella vulgaris* Algae

**DOI:** 10.3390/polym17060785

**Published:** 2025-03-15

**Authors:** Alexis López-Padilla, Misael Cortés-Rodríguez, Rodrigo Ortega-Toro

**Affiliations:** 1 Research Group on Applied Transformation of Industrial and Agro-Industrial Matrices (ITMIA), Food Engineering Department, Universidad de Cartagena, Avenida del Consulado Calle 30 No. 48–152, Cartagena de Indias 130015, Colombia; alopezp2@unicartagena.edu.co; 2 Functional Food Research Research Group (GAF), Department of Agricultural and Food Engineering, Faculty of Agricultural Sciences, Universidad Nacional de Colombia Sede Medellín, Cra. 65 No. 59A-110, Medellin 050034, Colombia; mcortesro@unal.edu.co; 3 Food Packaging and Shelf-Life Research Group (FP&SL), Food Engineering Department, Universidad de Cartagena, Avenida del Consulado Calle 30 No. 48–152, Cartagena de Indias 130015, Colombia

**Keywords:** microalgae in biopolymers, mechanical properties, optical properties, sustainable packaging

## Abstract

*Chlorella vulgaris* is a microalga with antioxidant and antimicrobial capacity that contains high levels of starch and proteins, essential for producing biodegradable packaging. This study aims to develop and characterize biofilms from soft wheat flour (SWF) and durum wheat flour (DWF) with yellow and white *Chlorella vulgaris*. The films were made using the compression molding method and characterized according to their physical, mechanical, and structural properties. The results indicated that yellow Chlorella films increase thickness and gloss and reduce water vapor permeability, which benefits applications requiring moisture retention. On the other hand, white Chlorella increases opacity and color saturation, making it suitable for less transparent packaging. Films with durum wheat and microalgae are stiffer and less elastic, while formulations with soft wheat and without microalgae are flexible. The surface texture is rougher in films with yellow Chlorella and more homogeneous in white Chlorella. These results suggest that *Chlorella vulgaris* allows biodegradable films to be tailored for specific applications in sustainable packaging.

## 1. Introduction

The development of biodegradable materials has increased in response to the growing need to reduce the environmental impact of conventional plastics. Among these new materials, biopolymer-based films have stood out for their potential to replace petroleum-derived plastics in packaging and coating applications.

Eco-friendly and biodegradable packaging is expected to significantly influence food quality and the development of the food industry and market in the coming years. The most common biopolymers used to manufacture biodegradable films in food packaging include polysaccharides and proteins, which come from natural and renewable sources, such as plants, animals, or microorganisms [1]. By 2026, starch blends and cellulose films are projected to lead production capacity in the sector. Furthermore, the agricultural area devoted to producing these renewable biopolymers is estimated to represent only 0.06% of the global total, indicating that their manufacturing will not significantly compete with the production of food or essential raw materials. This reinforces the viability of these materials as sustainable alternatives for industrial applications, including the production of biodegradable films [2].

Wheat flour, a widely available resource rich in polysaccharides and proteins, containing mainly starch (78–82%) and gluten (8–16%), is an adequate base to produce edible films and coatings [3]. Furthermore, using wheat flour to manufacture bioplastics represents a more economical and energy-efficient alternative than purified starch. This option provides attractive functional properties to the materials, although it has the disadvantage of having a lower breaking strength [4]. However, the incorporation of additional ingredients, such as microalgae, can improve their mechanical, structural, and functional properties [5], thus optimizing their performance in food packaging applications considering that they contain several bioactive molecules, such as polysaccharides, proteins, lipids (rich in omega-3 fatty acids), and pigments [6,7].

Microalgae have been highlighted for improving films’ mechanical properties and strength while providing them with antioxidant and antimicrobial characteristics. *Chlorella vulgaris* contains high levels of starch and proteins, essential for manufacturing edible coatings. This microalga has antioxidant and antimicrobial capacity [8,9]. *Chlorella vulgaris* is a microalga that contains lipids, carbohydrates, fiber, vitamins, and proteins but is especially interesting due to its protein content, which is usually up to 60% [10,11], with an exceptional nutritional profile, rich in beneficial compounds such as carotenoids (including β-carotene, lutein, and zeaxanthin), pigments, proteins with an optimal balance of amino acids, polyunsaturated fatty acids, minerals, and vitamins such as A, B12, E, and K. In addition, it is recognized as safe (GRAS) by the FDA in the USA and approved by the European Food Safety Authority. Also, the use of this microalga in biodegradable films has shown potential to improve key characteristics such as mechanical strength, water impermeability, and biodegradability, and due to its high content of proteins, carbohydrates, and fibers [8,12], as well as its natural and sustainable composition, its use as a raw material for biodegradable packaging is also being explored, representing an ecological alternative in industrial applications [13,14]. Furthermore, incorporating *Chlorella vulgaris* in its yellow and white variants allows for an exploration of the specific effects of each type of alga on the final properties of the films [14].

This study aims to develop and characterize biofilms from soft and hard wheat flours, evaluating the influence of the addition of yellow and white *Chlorella vulgaris* on the physical, mechanical, and structural properties. This research contributes to the formulation of sustainable films and offers relevant information for developing ecological alternatives in the packaging sector and other industrial fields.

## 2. Materials and Methods

### 2.1. Materials

Cartagena soft wheat flour (moisture 11.9%, total carbohydrates 73%, protein 12%, fat 1.4%, ash 1.7%) and semolina (moisture 15%, total carbohydrates 70%, protein 14%, fat 0.3%, ash 0%) were purchased from local chain stores in the city of Cartagena. Algae (yellow Chlorella and white Chlorella) were brought from All microalgae–natural products, SA 2445-413, in Pataias, Portugal. Yellow Chlorella had 9.5% fat, 55% carbohydrates, 9% fiber, and 32.5% protein, while white Chlorella had 8% fat, 24% carbohydrates, 23% fiber, and 30% protein. Finally, glycerol was supplied by PANREAC in Bogotá, Colombia.

### 2.2. Manufacturing of Soft Wheat and Durum Wheat Flour Films

To produce the films, the methodology proposed by Saiah et al. (2009) [15] was implemented with some modifications, where the compression molding method was implemented. To produce each film (soft wheat flour and durum wheat flour), flour and water were mixed in a proportion of 4:1; additionally, the different algae and 20% glycerol were added as a plasticizing agent concerning the amount of flour. Then, the components were homogenized in a FOOD MIXER model SL-B10 (Jiangmen Shengli Food Machinery Co., Ltd., Guangdong, China) until a uniform consistency was achieved. Table 1 shows the studied formulations. After 48 h, 10 g portions of the mixture were extracted to form the films, which were placed in a hydraulic press at 120 °C. These portions were subjected to different pressures ranging from 0 to 100 bar for approximately 2 min under each pressure.

### 2.3. Characterization of the Films

#### 2.3.1. Thickness

The thickness of the films was calculated with the help of a digital micrometer (TL268, Proster, Shenzhen, China). Seven random measurements of the films were made, and then the mean value and standard deviation were reported.

#### 2.3.2. Gloss

The gloss was determined at an angle of 60° according to ASTM D523 [16], using a gloss meter with a flat surface design (multi-angle gloss meter 3NH YG268, Minolta, Langenhagen, Germany). A total of six films were evaluated, and three measurements were taken of each sample. The results were expressed in luminance units (GU).

#### 2.3.3. Color

The color of the films was measured with a portable colorimeter (Colorimeter CHN Spec CS-10, CHNSpec Technology Co., Ltd., Zhejiang, China). This gave the CIE Lab* coordinates, the hue angle (h), and the chroma or saturation (c). For CIE Lab* coordinates, lightness is represented on the vertical axis, while the horizontal axes indicate the orientation towards colors such as red, green, blue, and yellow [17]. Finally, the color variation of a reference sample was determined. The color difference for each coordinate (L*, a*, and b*) was calculated to achieve this. The overall color distinction ΔE* was then determined by using Equation (1):

(1) $${∆E}^{*}=\sqrt{{∆L}^{*2}+{∆b}^{*2}+{∆a}^{*2}}$$

#### 2.3.4. Film Opacity and Internal Transmittance

A spectrophotometer (BIOBASE BK-UV1900PC UPR19G0005, BIOBASE Group, Shandong, China) was used to determine the opacity and transmittance of each film [18]. The opacity was calculated for each film using Equation (2):

(2) $$Opacity=\frac{Absorbance\;(600\;nm)}{Thickness(mm)}$$

The transmittance of the films was determined over the whole UV-Vis range in film samples (1 cm × 3 cm) equilibrated at 25 °C and 75% RH, within a wavelength range of between 200 and 1000 nm [19].

#### 2.3.5. Moisture Content and Water Solubility

The methodology proposed by Aguirre et al. (2013) [19] was followed to determine the moisture content and water solubility of the films. Film samples (2 cm × 2 cm) were first weighed (W0) and then dried in an oven at 60 °C until reaching a constant weight (W1). After drying, the samples were immersed in distilled water at a 1:10 ratio (film/water) for 24 h. Subsequently, the films were removed and dried again to a constant weight (W2), and corresponding Equations (3) and (4) were applied to calculate the moisture content and water solubility, respectively.

(3) $$\%\;Moisture=\frac{W1-W2}{W1}\times 100\%$$

(4) $$\%\;Solubility=\frac{W1-W2}{W1}\times 100\%$$

#### 2.3.6. Water Absorption Capacity

The water absorption capacity was quantified according to the protocol outlined by Pirsa (2021) [20]. The samples were meticulously prepared by sectioning them into 2 cm × 2 cm dimensions and placing them inside a desiccator containing calcium chloride, maintaining a humidity level of 0% at room temperature. The weights of the samples were recorded at 24 h intervals until a stable weight was reached, i.e., the mass of the dry film (Ws). After determining this stable weight, the samples were transferred to a desiccator containing a saturated potassium sulfate solution. In a manner analogous to the previous step, the samples were weighed every 24 h until the weight of the film reached equilibrium, this final weight being designated as the mass of the thoroughly wet film (Wh). Equation (5) was used to determine the water absorption capacity:

(5) $$\%\;Water\;absorption\;capacity=\frac{W_{h}-W_{s}}{W_{s}}\times 100\%$$

#### 2.3.7. Contact Angle in Water and Oil

A film sample (2 × 2 cm) was placed on a horizontal surface with a white background, and then a drop of water (with dye) or oil was deposited on the film’s surface. The image was captured with a digital camera after 30 s, maintaining a constant distance of 20 cm between the water or oil drop and the camera lens. Finally, the image obtained was analyzed using the Goniotrans software (version 1.0.3). This procedure was performed in triplicate for each formulation, and the mean value and standard deviation were reported [21].

#### 2.3.8. Water Vapor Permeability (WVP)

The evaluation was carried out using a gravimetric approach according to the methodology established by Aguirre et al. (2013), with several modifications [19]. The environmental parameters were manipulated to establish a humidity gradient ranging between 52.8% relative humidity and 100% relative humidity at a controlled temperature of 25 °C. Films free of physical imperfections were selected for the WVP evaluations. Payne-type permeation vessels filled with distilled water were used, allowing one film surface to be subjected to 100% relative humidity. These vessels and the films were placed in humidity-controlled cabinets at 25 °C, with the cabinet relative humidity maintained at 52.8% using supersaturated magnesium nitrate solutions. In addition, to improve the applicability of the films to products characterized by high water activity, the free surface of the film was exposed to a lower relative humidity environment during manufacturing. Vessels containing the films were subjected to systematic evaluation using a high-precision analytical balance with a sensitivity of 0.0001 g. After reaching a stable measurement condition, the water vapor transmission rate (WVTR) was calculated based on the slope of the regression line, delineating the decrease in weight over time. This value was then divided by the area of the film. This procedure was repeated three times, yielding results encompassing the mean value and the corresponding standard deviation.

#### 2.3.9. Mechanical Properties

This test was performed using the methodology proposed by Arrieta et al. (2024) [17]. The elastic modulus (EM), tensile strength (TS), and strain (E) of the analyzed films were determined. The experimental setup used a texture analyzer (TX-700, Lamy Rheology, Champagne, France) with a 500 N load cell, operating at a 50 mm/min rotation speed. The film samples measured 1 cm × 10 cm, with a clamping distance of 5 cm.

#### 2.3.10. Microstructure

The microstructural analysis of the cross-sections and surfaces of the films was carried out using a scanning electron microscope (JSM-5910, JEOL Ltd., Tokyo, Japan). The film samples were maintained in desiccators with P_2_O_5_ for 2 weeks to guarantee that water was not present in the sample. Film pieces, 0.5 cm^2^ in size, were cryofractured from films and fixed on copper stubs, gold-coated, and observed using an accelerating voltage of 10 kV.

#### 2.3.11. Statistical Analysis

The statistical analysis was carried out using an analysis of variance (ANOVA) methodology. Significant differences (*p* < 0.05) were evaluated using Fisher’s significant difference test between the various analyses performed on the samples. Statistical evaluations were performed using Statgraphics Centurion XVI software, version 16.2.04 (Manugistics Corp., Rockville, MD, USA).

## 3. Results and Discussion

### 3.1. Film Characterization

#### 3.1.1. Optical and Color Properties

Table 2 shows the optical and color properties of films made from soft wheat flour (SWF) or durum wheat flour (DWF) and additions of microalgae *Chlorella vulgaris* in their yellow and white variants. The formulations are evaluated for their brightness at 60°, color parameters (L*, a*, b*, chroma (C), and hue (h)), and total color difference (Δ*E*), providing a detailed insight into the visual appearance of the material. The data highlight the differences in lightness, saturation, and hue between the formulations, as well as the impact of the microalgae on the color variation.

The brightness varies between formulations, with SWF-Y being the brightest and DWF-W being the least bright. Films with white Chlorella (SWF-W and DWF-W) exhibit lower brightness levels (11.6 ± 1.7 and 7.3 ± 0.9, respectively) compared to yellow Chlorella. Among the combinations, durum wheat formulations (DWF-Y and DWF-W) have lower luminosity, showing that they are less precise than soft wheat ones. In general, films with white Chlorella (SWF-W and DWF-W) stand out for their high color saturation (chroma) and their intense yellow hue, while films from durum wheat flour (DWF) have lower brightness and clarity compared to soft wheat (SWF) and tend to be less green in hue. On the other hand, SWF-Y and DWF-Y present intermediate Δ*E* values, indicating that adding Chlorella, especially the white one, significantly affects color perception.

Rezaei and Chavoshizadeh (2020) [22], reported that the optical properties of the films were influenced by several factors, such as the internal structure of the polymer, the ability to mix different phases, the color of the added materials, the type of dispersion, and the size of the dispersed particles, as well as the thickness of the film.

#### 3.1.2. Physical and Water Absorption Properties

Table 3 presents the average values and the standard deviations of the physical and water absorption properties of the films developed from SWF and DWF, with and without the addition of *Chlorella vulgaris* microalgae (in its yellow and white variants). The parameters evaluated include film thickness, WVP, swelling index (Sw), moisture content (Xw), and water absorption capacity (Wca).

The results show significant variations in thickness, where formulations with microalgae, especially DWF-Y, present the highest thickness (404.7 μm), while SWF and DWF films without microalgae are the thinnest. This increase in thickness can be attributed to the composition of the added microalgae, as the high carbohydrate content in yellow Chlorella and the higher amount of fiber in white Chlorella probably provide structure and volume to the films.

Regarding WVP, formulations without microalgae (SWF and DWF) have higher values, indicating a higher vapor passing capacity compared to formulations containing Chlorella, such as SWF-Y and DWF-Y, which show lower WVP values. The presence of carbohydrates and proteins in yellow Chlorella could improve the water vapor barrier, which is beneficial for applications requiring lower permeability. Therefore, the presence of microalgae, especially yellow *Chlorella vulgaris*, can reduce water vapor diffusion due to its high carbohydrate and protein content, which favor a more compact matrix and reduce water mobility in the film structure. In addition, the interaction between the microalgae polysaccharides and the starch matrix can generate a denser network that hinders moisture transmission [23]. In this way, films formulated with *Chlorella vulgaris*, especially those with yellow Chlorella, present a more significant barrier to water vapor, making them suitable for moisture-sensitive products, such as nuts, biscuits, and baked snacks, as they would help prolong their crunchy texture.

The results of Sw indicate that formulations containing microalgae, particularly DWF-Y, exhibit a higher swelling capacity and suggest an increased interaction with water, likely influenced by the structural components of *Chlorella vulgaris*, such as its fiber and protein content. When comparing the results obtained with commercial polymers such as PLA or PCL, whose solubility values are 0.28 and 0.48 g of soluble film/g of dry film [24], it can be observed that the materials developed with wheat flour exhibit very similar values (ranging from 0.015 to 0.081 g solubilized film/g initial film), with even lower values for the hard wheat flour control. This may be attributed to the protein content in the hard wheat matrix and the strong interactions between protein and starch molecules in hard wheat compared to soft wheat.

On the other hand, the Wca remains relatively constant in all formulations, indicating that adding microalgae does not significantly affect this parameter. Finally, in Xw, the films with microalgae, especially DWF-Y, exhibit a higher value, indicating a higher water retention capacity than the formulation without microalgae (SWF and DWF).

Therefore, these data suggest that the incorporation of microalgae, particularly yellow Chlorella, increases the thickness, reduces water vapor permeability, and improves the water absorption capacity of the films, which could influence their applications as coatings or bioplastics [25].

#### 3.1.3. Internal Transmittance

**Figure 1:**
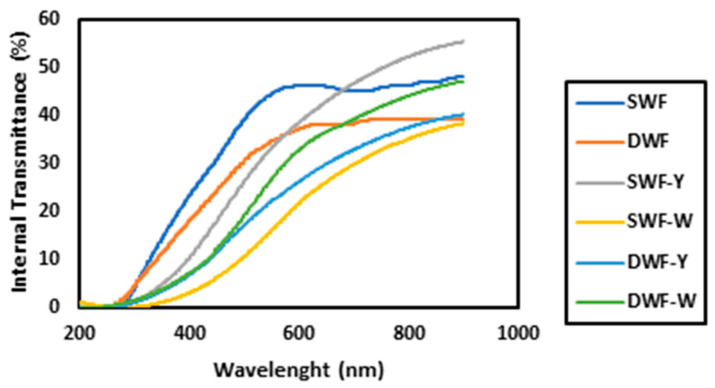
UV-Vis direct transmittance spectra of the different treatments.

Figure 1 shows the direct transmittance spectra in the UV-Vis range (200–1000 nm) of films made from different formulations of soft and hard wheat flour combined with two *Chlorella vulgaris* extracts (yellow and white). Internal transmittance is the amount of light passing through the films at different wavelengths [26].

Figure 1 shows that SWF films block UV light (200–400 nm) better than hard wheat flour (DWF) films, which is helpful for applications where UV protection is required. By adding *Chlorella vulgaris*, it is observed that the white variety (W) increases transparency in the visible range (400–700 nm), especially in the DWF-W formulation, which achieves the highest transmittance, making it the most transparent of all. In contrast, yellow Chlorella (Y) has a minor impact on transparency. Thus, formulations with white Chlorella would be suitable when greater clarity is sought, while soft wheat flour formulations without additives would be ideal for blocking UV light. On the other hand, it is observed that the SWF-Y film has the highest transmittance, which indicates that it is the most transparent, which coincides with the color data mentioned above, with SWF-Y being the brightest. Therefore, the presence of microalgae in films could provide protection against UV light, which would be beneficial for lipid-rich products, such as vegetable oils or chocolates, helping to prevent oxidation and prolong their shelf life.

**Figure 2:**
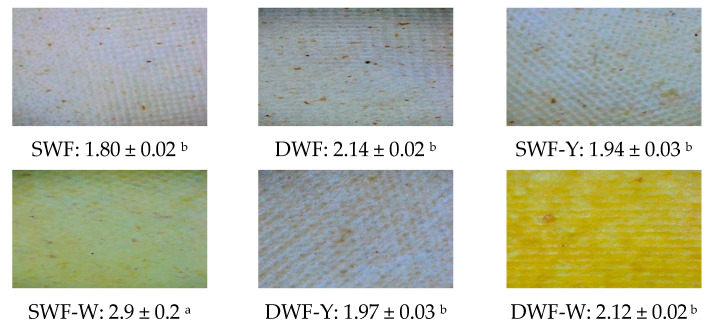
Film appearances from photographs and mean values and standard deviation of opacity of the films studied. Data are presented as mean +/− standard deviation and different superscript letters indicate significant statistical differences (*p* < 0.05) between formulations.

Figure 2 shows the developed films’ visual appearance, average opacity values, and standard deviation. Opacity is an essential parameter in the characterization of biodegradable films, as it affects their appearance and possible applications in packaging, where a transparent film may be desirable depending on the product to be packaged [26].

The opacity data reveal significant differences between the formulations. The SWF-W film presents the highest opacity value (2.92 ± 0.1), indicating lower transparency, and it is consistent with the high amount of fiber in white Chlorella, which could contribute to a denser structure and, therefore, higher opacity.

Films formulated with yellow Chlorella (SWF-Y and DWF-Y) and those without microalgae (SWF and DWF) show lower opacity values, in the range of 1.8 to 2.14, suggesting higher transparency. In the color data, these films also show lower saturation and lightness than films containing microalgae, consistent with their more transparent appearance. The lower opacity and saturation can be attributed to the absence of additional structural components from the microalgae, such as fiber and protein, resulting in a less dense structure. Therefore, there is a coherence between the opacity values and the color parameters of the films. Films with white Chlorella are the most opaque and present more saturated colors, while films without microalgae are more transparent and have less color intensity. The microalgae’s type and content affect not only the opacity but also the intensity and luminosity of the color, providing visual characteristics that could be adjusted according to the desired application [27]. On the other hand, the non-uniform distribution of *Chlorella vulgaris* in the films could be related to the interaction of components during the mixing and film-forming process, which may lead to localized accumulations of microalgae. The absence of yellow spots in SWF, DWF, and SWF-Y could be due to Chlorella pigments dispersed in the film matrix or to possible changes in its structure during thermal processing. On the other hand, the more prominent yellow hue in SWF-W and DWF-W compared to SWF-F and DWF-F may be associated with differences in the composition of the white Chlorella variant, especially in its pigment content, such as carotenoids or xanthophylls [28].

#### 3.1.4. Contact Angle

**Figure 3:**
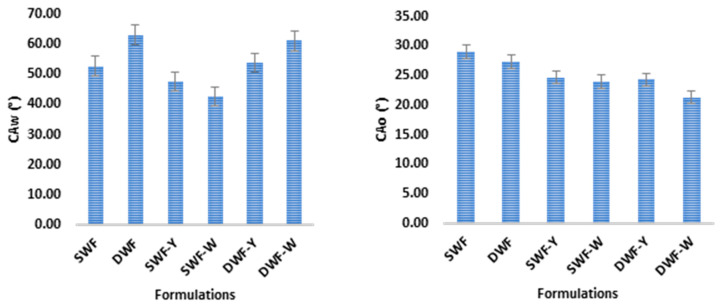
Mean values and standard deviation of the studied films’ water contact angle (CAw, °) and oil contact angle (CAo, °). Data are presented as mean +/− standard deviation.

Figure 3 compares the contact angles in water (CAw) and in oil (CAo) for different film formulations made from soft and hard wheat flours, as well as with additions of *Chlorella vulgaris* in two variants (yellow and white). Figure 3 suggests that the different formulations affect the hydrophobicity and oleophobic properties of the films, with notable differences in the behavior towards the water and a somewhat more homogeneous behavior towards oil. The additions of *Chlorella vulgaris* seem to influence the contact properties of the films.

In particular, the SWF films show that adding *Chlorella vulgaris* increases the hydrophobicity of the films (higher CAw). At the same time, its effect on oleophobicity (CAo) is low and indicates that Chlorella contributes more to repelling water than oil in the soft wheat films. In the case of DWF, adding *Chlorella vulgaris* also increases hydrophobicity (higher CAw) but has little effect on oleophobicity (CAo). As in the case of SWF, Chlorella affects the interaction with water more than with oil in the durum wheat films. Other studies revealed similar water contact angle values between 50 and 100°, where the high amylose film (64%) possessed a hydrophilic surface as it exhibited WCA θ < 90° [28].

**Figure 4:**
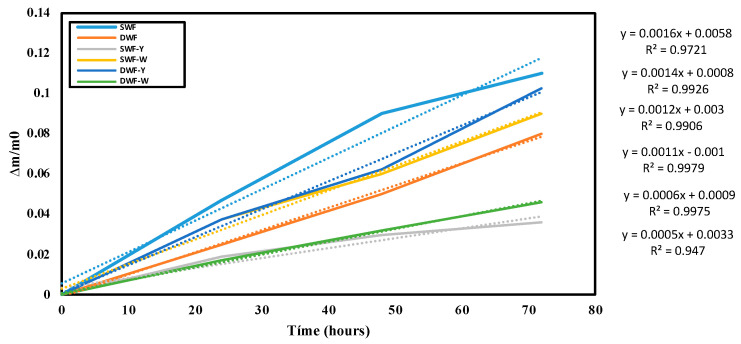
Cumulative weight gain of the films studied, stored at room temperature (25 °C). The dotted lines indicate trend lines.

Figure 4 shows the cumulative weight gain, indicating environmental moisture absorption or water retention.

Regarding the cumulative weight gain, SWF has the highest slope, indicating that it absorbs more moisture than the other formulations. Formulations containing yellow or white *Chlorella vulgaris* appear to reduce moisture absorption, particularly for durum wheat films with Chlorella, suggesting that the Chlorella additive might help control moisture gain in the films. Therefore, soft wheat films tend to absorb more moisture, and adding Chlorella (especially the white variant) appears to reduce this absorption, suggesting a barrier effect that could be beneficial in applications where moisture resistance is required.

**Figure 5:**
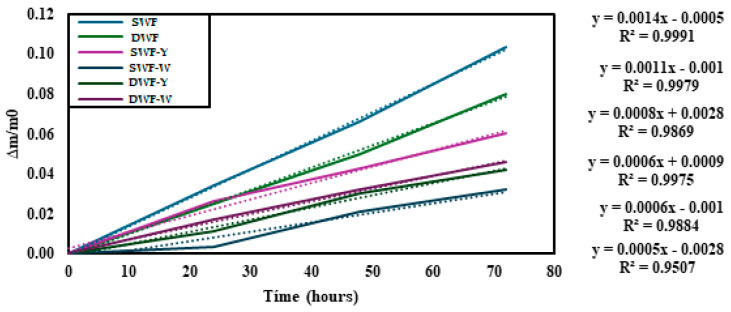
Cumulative weight loss of the studied films. The dotted lines indicate trend lines.

Figure 5 shows the cumulative weight loss of the different wheat flour film formulations. The data indicate that all films tend to lose weight over time.

Formulations with SWF show lower slopes in the weight loss curves, especially in the case of SWF-W, which presents the lowest cumulative weight loss. There is more excellent stability in weight retention under the conditions studied, which may be directly related to the materials’ water affinity and moisture retention capacity.

From the point of view of water affinity, soft wheat flour has a composition that favors interaction with water due to its content of carbohydrates, mainly starch and proteins, such as gluten, which can form three-dimensional networks that trap water molecules [29]. The incorporation of white Chlorella could amplify this capacity due to its high content of polysaccharides, lipids, and hydrophilic proteins, which have a recognized capacity to interact with water and stabilize moisture in food matrices. The synergy between soft wheat and white Chlorella can explain the lower slope observed in SWF-W. The latter could act as an agent that improves water retention by increasing the hydration capacity of the mixture. This would not only reduce the evaporation rate but could also limit the migration of water to the material’s surface, decreasing weight loss due to dehydration. Regarding moisture retention, the network structure formed by the flour components and white Chlorella will likely offer physical barriers that hinder the release of adsorbed and absorbed water. In addition, the polysaccharides present in Chlorella could act as wetting agents, retaining water in the capillaries and pores of the system, which explains the stability observed in the formulations with Chlorella [30,31,32,33].

#### 3.1.5. Mechanical Properties

Table 4 shows the mean values and standard deviations of the mechanical parameters such as tensile strength (TS), strain (E), and Young’s modulus (EM) of the films studied. These parameters reflect the strength, elasticity, and stiffness properties of the different film formulations.

TS values indicate the maximum strength of the films before breaking. Formulations containing DWF combined with microalgae (especially DWF-Y and DWF-W) present the highest tensile strength values (10.7 to 11.2 MPa), suggesting higher strength compared to films made with soft wheat flour alone (SWF: 7.6 MPa). This may be due to the more structured composition and additional components of microalgae, especially the high fiber and protein content in *Chlorella vulgaris*. Therefore, the fiber in white *Chlorella vulgaris* can also act as a reinforcement, distributing itself within the polymeric matrix and favoring better tensile strength. Also, the possible interaction between the protein components and the polysaccharides of the film can generate additional bonds that strengthen the structure of the material [34]. On the other hand, strain measures the ability of films to elongate before breaking, i.e., their flexibility. SWF films (soft wheat flour without microalgae) present the highest deformation value (140%), indicating that they are more elastic or flexible, like other studies indicating E (%) is 121 and TS is 5.31 Mpa for corn starch films [35]. In contrast, formulations with white Chlorella and yellow Chlorella (especially DWF-W with a value of 90%) show a lower deformation, indicating that these films are less elastic and more rigid. The lower elasticity could be a consequence of the greater rigidity provided by the structural components of the microalgae. The Young’s modulus is a measure of the rigidity of the films. The highest EM values correspond to the formulations with durum wheat flour and microalgae (especially DWF-W: 50.2 MPa), indicating that these films are more rigid and less flexible. Films with soft wheat flour without microalgae (SWF) have the lowest Young’s modulus value (27.2 MPa), suggesting that they are less stiff and more compliant. The higher stiffness in formulations with microalgae can be attributed to the structural effects of the fiber and protein content in microalgae, which reinforce the film structure. Similar studies indicated that films with higher starch content exhibited a higher elastic modulus and tensile strength considering the type of chitosan used: polymer–plasticizer and polymer–solvent interactions prevailed and led to the development of more flexible materials in film formulations with low-molecular-weight chitosan [35]. Other studies suggest that the mechanical properties of hydrolyzed corn starch-based films were improved due to the enzymatic hydrolysis process, considering that it breaks starch molecules into smaller units, resulting in greater chain flexibility, better film-forming capacity, and stronger intermolecular bonds [36,37].

Therefore, DWF-W and DWF-Y films are stronger and stiffer, although less elastic, compared to formulations made with soft wheat flour (SWF) alone. This suggests that the type of flour and the inclusion of microalgae not only increase the mechanical strength but also decrease the flexibility of the films. These mechanical properties could make formulations with microalgae more suitable for applications requiring higher strength and stiffness, while formulations without microalgae may be preferable in applications requiring higher flexibility. Future research should focus on the long-term stability of films under different storage conditions, such as temperature and humidity variations. Evaluating the factors affect their mechanical, barrier and biodegradability properties will determine their suitability for commercial applications.

#### 3.1.6. Microstructure

**Figure 6:**
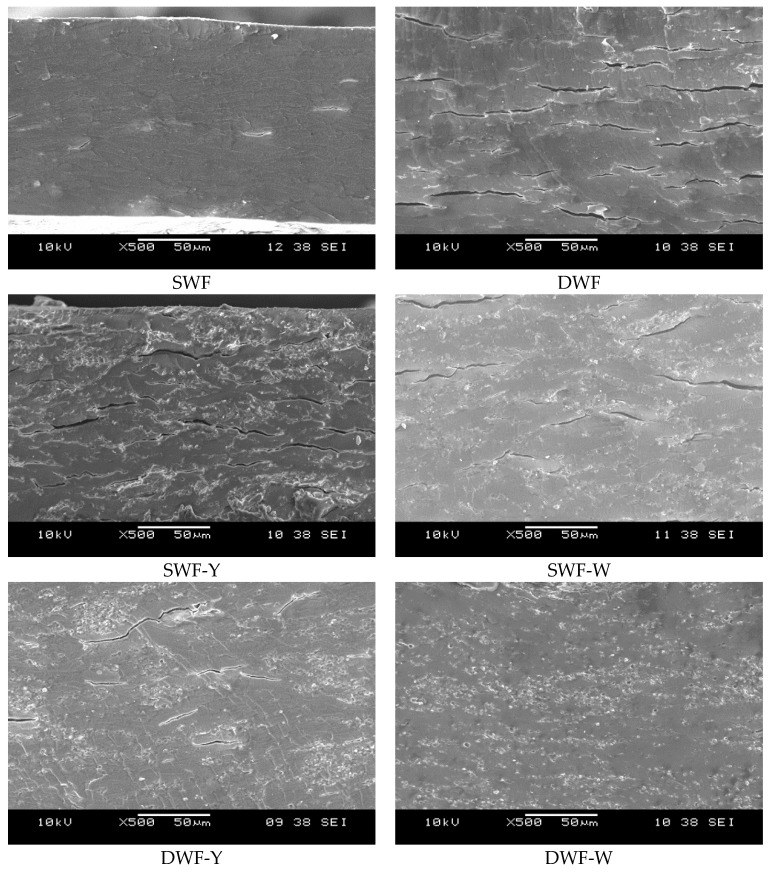
Cross-section SEM micrographs of the studied films at 500×.

Figure 6 shows a microstructural analysis of the biodegradable films using a scanning electron. SEM images show differences in the microstructure of biodegradable films depending on their formulation. Soft wheat flour (SWF)-based films tend to be more homogeneous, while DWF-based films have a rougher texture with more significant cracks. The addition of *Chlorella vulgaris* (both yellow and white) modifies the morphology, generating more irregular and heterogeneous surfaces, especially in DWF-Y and DWF-W films. Films with Chlorella seem to show greater porosity and lower structural cohesion compared to formulations without microalgae, which could influence their mechanical properties and biodegradability.

Similar studies with wheat flour have shown that the films have a relatively smooth surface without cracks or holes. However, with increasing protein content, an increase in roughness is observed, possibly due to protein denaturation during starch gelatinization, which favors a better interaction between starch and water during cooling [3,38,39,40].

From the mechanical properties point of view, the microstructure observed in SEM agrees with the values of tensile strength (TS), strain (%E), and Young’s modulus (EM). The greater homogeneity of the SWF films without microalgae is reflected through higher elasticity (140% elongation), which indicates a better cohesion of the polymeric matrix. In contrast, the greater roughness, and cracks in the films with DWF, especially those with microalgae, are associated with increased stiffness and mechanical strength, as demonstrated by the higher Young’s modulus (up to 50.2 MPa in DWF-W). The lower flexibility in these formulations suggests that the more heterogeneous and porous structure hinders the mobility of the polymeric chains, resulting in more rigid films. Regarding barrier properties, the observed microstructure also explains the differences in water WVP. Films with more homogeneous and less porous surfaces (SWF and DWF) present a higher water vapor permeability, indicating that the distribution of polymers in the matrix allows a greater diffusion of water molecules. In contrast, incorporating Chlorella, particularly yellow Chlorella, improves the water vapor barrier, reducing the WVP. This could be due to the presence of proteins and carbohydrates in the microalgae, which increase the density of the matrix and decrease the ease of passage of water vapor through the material.

**Figure 7:**
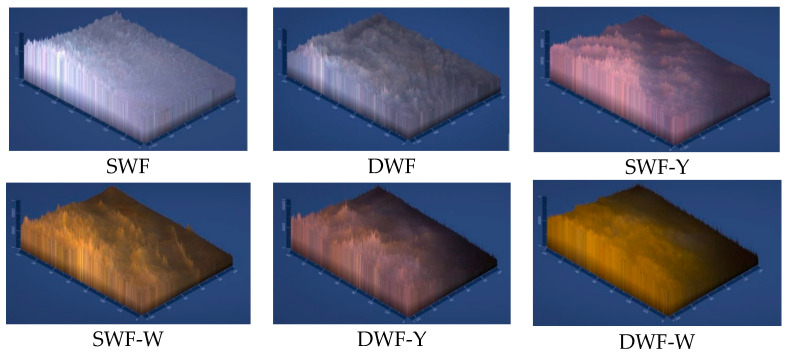
The 2.5D (10×) surface optical micrographs of the biodegradable films studied.

Figure 7 shows 2.5D micrographs of the films studied, allowing the variation in surface roughness and elevation to be observed. The basic formulations without algae (SWF and DWF) present a relatively homogeneous surface with less variation in elevation. However, by adding yellow Chlorella (in SWF-Y and DWF-Y), the surface acquires a rougher and raised texture, indicating greater heterogeneity, possibly attributed to the high carbohydrate and protein content in this Chlorella variant. In the formulations with white Chlorella (SWF-W and DWF-W), although roughness is also observed, the texture seems somewhat more uniform compared to yellow Chlorella, which may be related to its higher fiber content and lower proportion of carbohydrates.

## 4. Conclusions

This study demonstrates that the addition of *Chlorella vulgaris* to biodegradable films based on SWF and DWF allows for fine-tuning of their optical, barrier, and mechanical properties, offering versatile options for sustainable applications. Formulations with yellow Chlorella (SWF-Y and DWF-Y) have a greater thickness, gloss, and lower water vapor permeability, which improves their capacity as a moisture barrier. These properties make them especially suitable for applications that require water retention and lower permeability. On the other hand, formulations with white Chlorella (SWF-W and DWF-W) stand out for their high opacity and color saturation, which give them an intense yellow hue and a less transparent appearance, characteristics that could be desirable in packaging where direct exposure to the content is sought. Soft wheat films block UV light better, and when supplemented with white Chlorella, they exhibit higher stability in terms of weight retention and lower moisture absorption, which are advantageous in conditions requiring moisture resistance.

Regarding mechanical properties, films with microalgae, especially those formulated with durum wheat (DWF), show higher stiffness and lower elasticity, suggesting a stronger and less flexible structure. This is reflected in the high Young’s modulus values, particularly in DWF-W, which achieves the highest stiffness. Formulations without microalgae and with soft wheat flour are the most flexible, which can be beneficial for applications requiring conformability and flexibility. Yellow Chlorella reveals a rougher surface texture in the films due to its high carbohydrate and protein content, while films with white Chlorella exhibit a more homogeneous texture, attributable to its higher fiber content. Thus, the incorporation of *Chlorella vulgaris*, either in its yellow or white variant, allows the structure, resistance, and functionality of the films to be modified, which opens the door to its use in a variety of applications in biodegradable packaging, where properties such as opacity, permeability, and resistance can be adjusted according to the specific requirements of the final product. Future research could explore other microalgae or biopolymers, such as PLA or PHA, and test the films under actual packaging conditions. In addition, it will be essential to evaluate scalability, production costs, and decomposition in different environments to ensure their commercial viability in the sustainable packaging market and explore alternative processing methods.

## Figures and Tables

**Table 1:** Formulations of the soft and hard wheat flour films studied.

Formulations	Soft Wheat Flour	Durum Wheat Flour	Yellow *Chlorella vulgaris*	White *Chlorella vulgaris*
SWF	1.0	0.0	0.0	0.0
DWF	0.0	1.0	0.0	0.0
SWF-Y	1.0	0.0	0.25	0.0
SWF-W	1.0	0.0	0.0	0.25
DWF-Y	0.0	1.0	0.25	0.0
DWF-W	0.0	1.0	0.0	0.25

**Table 2:** Mean values and standard deviation of the brightness (GU) and color parameters (lightness (L*), red/green (a*), yellow/blue (b*), chromaticity (C), and hue angle (h, °)) of the studied films.

Formulations	Gloss at 60°	Color Parameters					Δ*E*
		L*	a*	b*	C	h	
SWF	9.3 ± 1.6 ^a^	31.1 ± 0.3 ^a^	−5.6 ± 0.1 ^b^	3.3 ± 0.1 ^f^	6.2 ± 0.3 ^f^	149.8 ± 0.6 ^a^	-
DWF	8.6 ± 0.9 ^a^	35.3 ± 1.2 ^b^	−3.6 ± 0.4 ^c^	8.4 ± 0.2 ^e^	24.1 ± 0.01 ^e^	78.7 ± 1.3 ^b^	-
SWF-Y	12.8 ± 0.5 ^ab^	54.1 ± 0.5 ^bc^	−4.0 ± 0.2 ^b^	16.7 ± 0.5 ^d^	17.1 ± 0.4 ^d^	95.0 ± 0.7 ^c^	9.9 ± 0.5 ^d^
SWF-W	11.6 ± 1.7 ^b^	53.0 ^a^ ± 0.9 ^cd^	−3.7 ± 0.2 ^b^	40.3 ± 0.5 ^b^	40.5 ± 0.5 ^b^	95.3 ± 0.3 ^c^	33.3 ± 1.1 ^b^
DWF-Y	7.4 ± 0.9 ^c^	50.5 ^a^ ± 0.4 ^e^	−2.5 ± 0.6 ^a^	22.7 ± 0.8 ^c^	22.9 ± 0.7 ^c^	96.3 ± 1.6 ^c^	16.5 ± 0.6 ^c^
DWF-W	7.3 ± 0.9 ^c^	52.6 ± 0.6 ^d^	−3.5 ± 0.2 ^c^	42.8 ± 1.0 ^a^	42.9 ± 1.0 ^a^	94.7 ± 0.1 ^c^	35.6 ± 1.7 ^d^

Data are presented as mean +/− standard deviation. Different superscript letters indicate significant statistical differences (*p* < 0.05) between formulations.

**Table 3:** Mean values and standard deviation of thickness (μm), water vapor permeability (g-mm/kPa-h-m^2^), swelling index (g solubilized film/g dry film), moisture content (g water/g dry film), and water absorption capacity (g dry film/g wet film) of the films studied.

Formulations	Thickness (μm)	WVP	Sw	Wca	Xw
SWF	177.8 ± 0.02 ^c^	4.14 ± 0.02 ^a^	0.034 ± 0.08 ^b^	0.37 ± 0.03 ^a^	0.147 ± 0.22 ^a^
DWF	183.5 ± 0.02 ^b^	4.20 ± 0.06 ^a^	0.015 ± 0.15 ^c^	0.40 ± 0.03 ^a^	0.130 ± 0.42 ^a^
SWF-Y	199.4 ± 0.04 ^e^	0.04 ± 0.01 ^c^	0.028 ± 0.17 ^b^	0.41 ± 0.08 ^a^	0.223 ± 1.11 ^b^
SWF-W	297.7 ± 0.05 ^cd^	0.05 ± 0.02 ^bc^	0.027 ± 0.25 ^b^	0.40 ± 0.02 ^a^	0.215 ± 0.46 ^b^
DWF-Y	404.7 ± 0.02 ^a^	0.04 ± 0.01 ^bc^	0.081 ± 0.31 ^a^	0.36 ± 0.07 ^a^	0.272 ± 1.15 ^c^
DWF-W	273.0 ± 0.02 ^d^	0.11 ± 0.07 ^b^	0.029 ± 0.17 ^b^	0.39 ± 0.02 ^a^	0.220 ± 0.84 ^b^

Data are presented as mean +/− standard deviation. Different superscript letters indicate significant statistical differences (*p* < 0.05) between formulations.

**Table 4:** Mean values and standard deviation of the mechanical parameters, strain (E), stress (TS), and Young’s modulus (EM), of the films studied.

Formulations	TS (Mpa)	E(%)	EM (Mpa)
SWF	7.6 ± 0.2 ^d^	140 ± 2 ^a^	27.2 ± 0.6 ^e^
DWF	8.8 ± 0.3 ^c^	121 ± 2 ^b^	31.7 ± 0.5
SWF-Y	9.1 ± 0.4 ^bc^	115 ± 3 ^c^	40.2 ± 0.5
SWF-W	9.5 ± 0.2 ^b^	100 ± 3 ^d^	45.0 ± 0.4
DWF-Y	10.7 ± 0.2 ^a^	105 ± 2 ^d^	45.5 ± 0.4
DWF-W	11.2 ± 0.3 ^a^	90 ± 2 ^e^	50.2 ± 0.3

Data are presented as mean +/− standard deviation. Different superscript letters in the columns indicate significant differences (*p* < 0.05) between formulations.

## Data Availability

Data supporting the results of this study are available upon request from the corresponding author.
